# A review for DFT in chemical mechanism of SERS studies

**DOI:** 10.1098/rsos.242000

**Published:** 2025-06-04

**Authors:** Aleksandr Zozulya, Andrey Zyubin, Ilia Samusev

**Affiliations:** ^1^ Immanuel Kant Baltic Federal University, Kaliningrad, Russia

**Keywords:** surface enhanced Raman scattering, nanoparticles, chemical mechanism, density functional theory

## Abstract

This review presents the state of the art in using density functional theory (DFT) to investigate the mechanism of chemical enhancement in surface enhanced Raman scattering (SERS). Computational DFT models have been shown to align well with experimental data and to be useful in their interpretation. In this context, the combined use of theoretical data and experimental SERS results can help explore the mechanisms contributing to chemical amplification. This review examines the application of DFT to estimate chemical enhancement of SERS under the following conditions: the presence of silver ions on the surface, the size and stability of metal clusters, the energy characteristics of the investigated molecule in the system from the cluster size in molecule-nanoparticle models, changes in the spatial orientation of the molecule on the nanoparticle surface depending on the concentration of molecules. Additionally, the review analyses the influence of the metal cluster shape and size in DFT calculations in simplified cluster systems. This information will be useful for researchers working with experimental SERS aspects.

## 1. Introduction

The density functional method is currently the most widely used and successful approach for calculating the molecular structure parameters of substances. It is based on the Hohenberg–Kohn theorem, which states that the properties of interacting particles in a system can be determined using the electron density functional.

By applying the Kohn–Sham formalism to the energy equation derived from the Hartree–Fock theory, the following equation can be obtained:


(1.1) $$E=V\;+\;\left\langle hP\right\rangle \;+\;\frac{1}{2}\left\langle PJ\left(P\right)\right\rangle \;+\;E_{X}\left[P\right]\;+\;E_{C}\left[P\right],$$

where *V* is the nuclear repulsion energy, ⟨hP⟩ stands for one-electron energy, $\frac{1}{2}\langle PJ(P)\rangle$ is a classical Coulomb repulsion of electrons, $E_{X}[P]$ represents energy exchange functional, $E_{C}[P]$ is the correlation energy functional, and the electron density has the following form [1–4]:


(1.2) $$P\left(r\right)=\sum_{i}^{N}\left|\varphi_{i}\left(r\right)\right|\;.$$

This approach greatly simplifies calculations by reducing computational time with no loss to the quality of the results. Density functional theory (DFT) methods are widely used across a variety of fields, including chemistry, catalysis, surface science, electrochemistry and optics. Thus, the authors of [5] modelled three dyes—G188, G268 and G270—to investigate the efficiency of their performance from the change of π-conjugation fragment in the structure of the compounds. They were able to demonstrate that elongating π-spacer moiety can effectively modulate photoabsorption properties, TiO_2_ conduction band shift and interface charge recombination. In addition, for G270 (the dye with the longest π-conjugation), selecting an appropriate substituent in the π-spacer allowed for improved coupling characteristics. Other groups of researchers [6,7] focused on studying ligands (some of which have carboxyl groups and others are alkoxysilanes) that are used to anchor dyes to the NiO surface.

**Figure 1.**
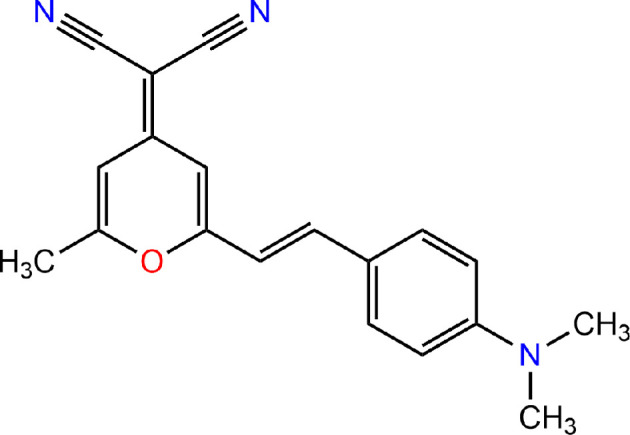
Molecular structure of the pyran dye D2-Me.

DFT and time-dependent (TD)-DFT calculations can be performed for several pyran dyes [8]. According to the calculation results available, the most effective donor dye is 4-(dicyanomethylene)−2-methyl−6-(p-dimethylaminostyryl)−4H-pyran (D2-Me) (figure 1).

**Figure 2.**
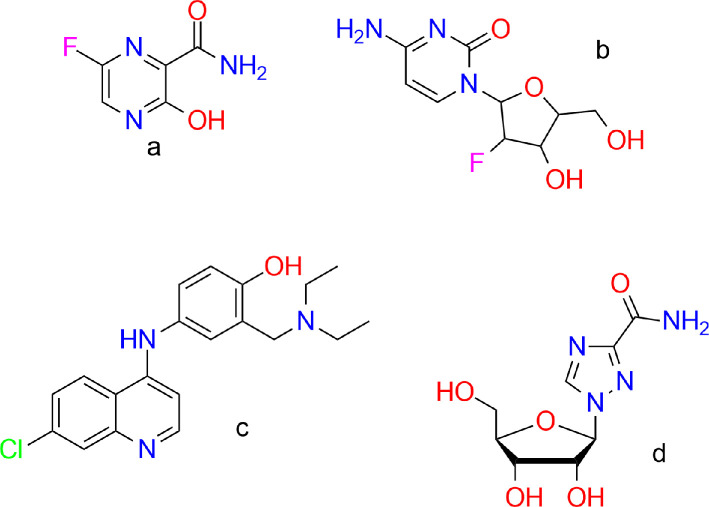
(a) Favipiravir, (b) 2′-fluoro−2′-deoxycytidine, (c) amodiaquine and (d) ribavirin.

Another important area of modelling application is drug development. In one study [9], various characteristics of topiroxostat, including potential and kinetic energy and dipole moment, were calculated. Its electrophilic and nucleophilic centres were also identified, which may help predict possible reactions with metabolites in the body. In another study [10], parameters such as electronegativity, chemical potential, universal hardness, universal softness, universal electrophilicity index and absolute softness were calculated for sulfonamide and its complexes with metals. These parameters are important for predicting biological activity of the compound (toxicity and reactivity). In [11], four drugs—favipiravir, amodiaquine, 2'-fluoro−2'-deoxycytidine and ribavirin—were investigated as COVID−19 inhibitors using DFT and molecular docking. The study found that amodiaquine may be the most promising drug due to its highest affinity for the receptor. This could be attributed to the fact that the compound can act as an electron donor (due to highest occupied molecular orbital (HOMO)), to the presence of electrophilic centres, as well as to high basicity and dipole moment (figure 2).

In [12], several sulfamide-based compounds were obtained in complex with metals. DFT modelling was used to characterize the substances, indicating that they may be bioactive. In the calculations, this was indicated by molecular descriptors, electrostatic potential capacitance, boundary studies of molecular orbitals, the single-electron transfer process, energy gap and electron injection ability. Another group [4] used DFT calculations to design polymers for drug delivery applications. The DFT method can also be applied to study other important properties of substances, such as adsorption. For example, the authors of [13] calculated the influence of functional groups on carbon sorbents’ ability to adsorb various dyes. Additionally [7] investigated the adsorption of paprika dye on the TiO_2_ surface for dye-sensitized solar cells to understand the interaction between matter and semiconductor.

**Figure 3.**
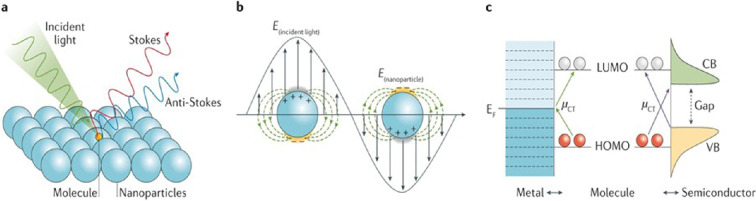
Enhanced Raman scattering of a molecule adsorbed onto nanostructured particles resulting in emitted radiation with a lower (red) or higher (blue) frequency than the incident light, known as Stokes and anti-Stokes scattering, respectively (a). Localized surface plasmon resonance contribution to surface-enhanced Raman spectroscopy (SERS), electrical field (*E*); an enhanced electrical field on the metal surface allows Raman signal amplification (b). Charge transfer (CT) contribution to SERS at a metal–molecule or semiconductor–molecule interface; the CT transition (*µ*CT) and arrows (green: metal–molecule; purple: semiconductor–molecule) show the CT directions. Red and white circles represent molecular orbitals (c). CB, conduction band; EF, Fermi level; HOMO, highest occupied molecular orbital; LUMO, lowest unoccupied molecular orbital; VB, valence band [15].

Surface-enhanced Raman spectroscopy (SERS) phenomenon [14] significantly enhances the Raman response of molecules adsorbed on nanostructured metal surfaces, such as silver, gold, platinum and rhodium. This phenomenon allows for repeatedly amplifying the Raman signal and is a superposition of electromagnetic and chemical mechanisms (figure 3).

To realize the first mechanism, two conditions must be met: (i) the substrate must consist of a metal with high optical reflectivity, meaning that the imaginary part of the dielectric permittivity must be very small in the spectral region of Raman excitation; and (ii) the wavelength of the excitation radiation must fall within the plasmon band of metal nanoparticles so that the molecules adhering to the metal are in the region of the excited surface plasmon. Under these conditions, the Raman signal can be amplified by a factor of 10^10^−10^15^ [16–18], allowing the detection of the spectra of molecular submonolayers adsorbed on nanostructured metals without requiring the formation of chemical bonds between molecules and the metal. The second contribution to SERS enhancement observed in the chemisorption of the molecule at active sites on the metal surface leads to a chemical mechanism that depends on the change in molecular polarizability due to the formation of metal−molecule complexes. As a result, the Raman spectra of chemisorbed particles differ from those of the corresponding free molecules [19]. While the electromagnetic contribution is essential for the SERS effect, the chemical contribution, though it only enhances the Raman signal by a factor of up to 100, plays a key role in determining the spectral picture. This is because the formation of surface complexes leads to significant frequency shifts and intensity changes of SERS bands compared with those observed in the conventional Raman spectra of unadsorbed molecules. This chemical enhancement mechanism can be divided into three parts. The first is ground-state chemical enhancement, which arises from changes in the chemical environment around the target molecule. The second is resonance Raman amplification, caused by resonance between the molecular transition and the excitation wavelength. The third is charge transfer, which results from the resonance between the new transition between adsorbed molecules and the excitation wavelength. Among these, charge transfer between molecules and metals serves as the most direct evidence of chemical enhancement [20]. To evaluate this contribution, DFT methods are used to analyse Raman shifts that occur during adsorption. This helps clarify the nature of the interaction in the ligand–nanoparticle binding region, carry out spectral identification of such regions and register changes in the structure of molecules in the binding centres. Additionally, DFT makes it possible to estimate the energy parameters of the complex and the corresponding relative intensities of Raman modes.

The aim of this review is to demonstrate how DFT modelling can be used in the study of the chemical mechanism of SERS, how theory is consistent with experimental results and how the constructed nanoparticle model affects the outcome.

## 2. Application of density functional theory in the study of the chemical mechanism of surface enhanced Raman scattering

**Figure 4.**
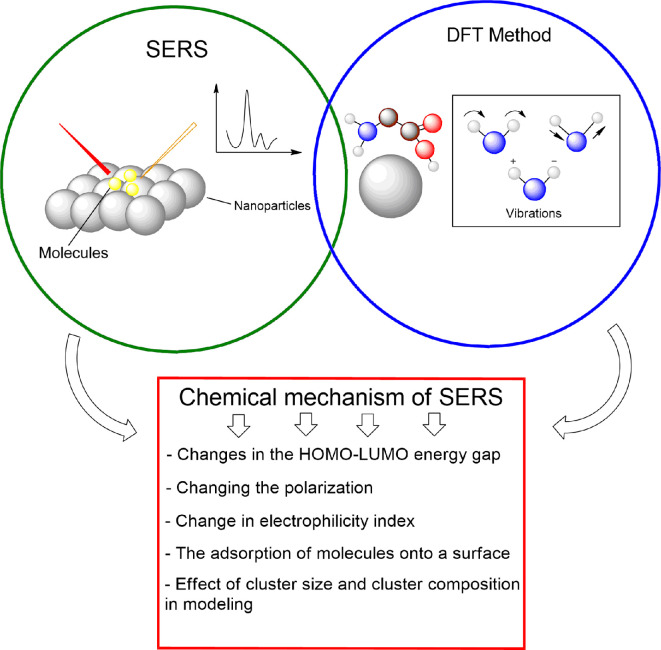
A schematic outline for a review article.

The role of chemical amplification in SERS can be investigated by analysing several parameters (figure 4). For example, changes in the HOMO–lowest unoccupied molecular orbital (LUMO) energy gap, which in turn affects other properties like electrophilicity.

This parameter helps explain the kinetic stability and reactivity of a molecule. The smaller the energy value of the forbidden zone, the higher the reactivity of the compound. Another important parameter is the change in the intensity of peaks by vibrational groups of atoms in the Raman spectrum. This characteristic makes it possible to understand through which functional group a molecule binds to a nanoparticle, how it is located relative to the metal surface, and how the concentration of the substance analysed affects it. For example, as shown in [21], when the 2-trifluoroacetylpyrrole (TFAP) molecule binds to gold and silver clusters, its energy gap decreases from the value of 4.65 (in the absence of metal) to 1.83 and 0.73 for complexes with Au_4_ and Ag_3_, respectively. Additionally, an increase in the electrophilicity index value of TFAP, TFAP-Ag_3_ and TFAP-Au_4_ systems was observed as a result of the interaction of the molecule with the nanoparticles; the calculated values were 4.89, 16.97 and 11.66 eV, respectively. The significantly higher electrophilicity index of TFAP-Ag₃ and TFAP-Au₄ compared with TFAP alone indicates that the molecule accepts electrons from the metal clusters. Furthermore, the enhanced peak intensity of the carbonyl group C=O, as well as C–C, N–H and CH vibrations in the pyrrole ring on the Raman spectrum, mean that chemisorption on the clusters occurred in an oblique orientation through the oxygen atom in the carbonyl group, and the nitrogen atom in the pyrrole ring. Similar calculations were performed for other organic molecules. For methyl−4-bromopyrrol−2-carboxylate, the energy gap decreased from 5.23 to 1.20 eV for the Ag_3_ cluster, and to 2.65 eV for Au_4_ [22]. For 2-(trichloroacetyl) pyrrole, the HOMO–LUMO gap decreased from 4.59 to 0.73 eV for Ag_3_, and to 1.92 eV for Au_4_ [23]. For 1-methylpyrrole−2-carbonyl chloride (MPCC) [24], the energy gap went from 5.05 to 0.79 eV and to 2.43 eV for complexes with Ag_3_ and Au_4_, respectively.

**Figure 5.**
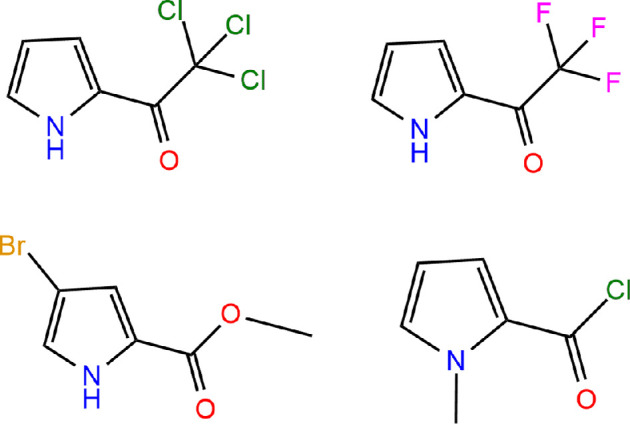
2-(trichloroacetyl)pyrrole, 2-(trifluoroacetyl)pyrrole, methyl−4-bromopyrrol−2-carboxylate, 1-methylpyrrole−2-carbonyl chloride.

Chemisorption of the first compound occurred through the pairs of electrons of the oxygen atom in the carbonyl group C=O, the π system of the pyrrole ring and the lone pairs of electrons of the bromine atom. For the second molecule, adsorption took place via the pair of electrons of the oxygen atom in the carbonyl group, the lone pair of electrons of the chlorine atom in the trichloro group, and the π system of the pyrrole ring. According to calculations, MPCC has a stronger affinity for gold nanoparticles when binding to the oxygen atom rather than the nitrogen atom in the pyrrole ring or the chlorine atom. However, similar to the previous compounds, this molecule is generally characterized by chemisorption to silver and gold nanoparticles through the oxygen atom in the CO functional group, π-system of the pyrrole ring and chlorine. The last three compounds, along with 2-trifluoroacetylpyrrole, were also in an inclined orientation relative to the nanoparticle surface. The molecular structures of all four compounds are shown in figure 5.

The study [25] also showed a change in the energy gap following the binding of alectinib to an Au_6_ cluster. Similarly, another study [26] observed a reduction in the energy gap from 4.66 to 3.02 eV after the adsorption of N-butyl−2-isonicotinoylhydrazine−1-carboxamide (INC) on a metal surface. Thermodynamic parameters (ΔE, ΔH, ΔG and ΔS) were calculated, yielding values of −6.84, −5.41, −6.51 and −0.90 eV molK^−1^, respectively. The polarizability of INC increased from 157.15 to 463.99 atomic units upon adsorption with Ag_6_, and the dipole moment increased from 4.31 Debye (INC) to 4.38 Debye (INC-Ag_6_). The changes in thermodynamic parameters are negative, which suggests that the adsorption process is spontaneous. A change in electrophilicity index was also observed, increasing from 3.51 eV (INC) to 4.56 eV (INC-Ag_6_), indicating that INC tends to become more electrophilic in the presence of Ag and that the molecule prefers to withdraw electrons from the metal cluster. Additionally, it has been found that the energy gap depends on the cluster size. In [27], the researchers demonstrated that with the increase in the number of atoms in the cluster from one to three, the value of the forbidden zone decreases twofold. At the same time, increasing the cluster size to six atoms led to an increase in the energy gap. Further calculations showed that clusters with an even number of gold atoms exhibited the largest energy reduction, while no such dependence was observed for silver clusters. This issue will be discussed in greater detail in the next chapter. In [28], it was demonstrated that the binding of fenbendazole to a cluster of gold nanoparticles led to a decrease in energy gap between LUMO and HOMO. Specifically, upon binding to an Au_3_ cluster, the energy gap decreased from 4.66 to 2.12 eV.

**Figure 6.**
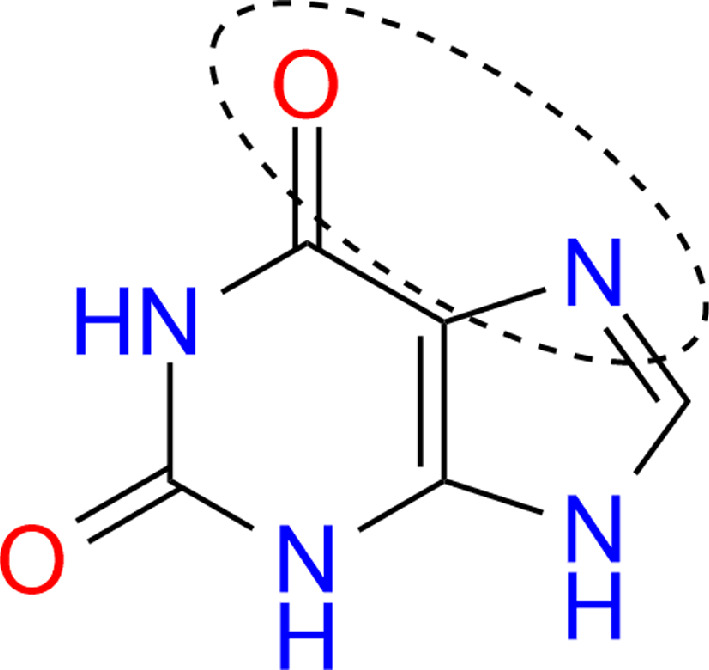
Xanthine.

In the same study, the location of fenbendazole relative to the nanoparticle was investigated by analysing differences in relative intensities between Raman spectra and SERS spectra, where a shift of peaks was observed. DFT calculations attributed this shift to the vertical adsorption of the molecule on the surface. The strong interaction between fenbendazole and gold nanoparticles is due to the imidazole approach. The chemical bonding and gold on imidazole exhibit a strong charge transfer effect. Another paper [29] examined the adsorption of molecules onto a gold surface, although it focused on simpler molecular structures. Specifically, nitrobenzene/aniline molecules were found to vertically adsorb on the surface of gold nanosphere (AuNPs) via nitro- or amino group, forming molecular complexes. In [30], the binding of the molecule (L-cysteine) to the gold cluster was investigated by comparing SERS spectra obtained by both calculated and experimental methods. In [31], a comparison between the calculated Raman spectra and the SERS spectra of thione, thiol and thiolate tautomers revealed that 2-thiazolin−2-thiol exists predominantly in the form of a thiol conformer on the surface of Ag and Au nanoparticles. Thanks to DFT calculations, researchers in [32] were able to identify the characteristic bands of xanthine SERS, including the tautomer that binds to the nanoparticle, and determine the groups through which xanthine binds to the metal surface, namely oxygen in the carbonyl group and nitrogen−7 atom in the imidazole fragment of purine. They were also able to establish that chemisorption occurs with charge transfer from the molecule to the metal (figure 6).

In [33], a study of the interaction between copper and pyridine (Cu–Py) revealed a dependence on the distance of the nitrogen atom N of the molecule to the surface of the Cu-N nanoparticle. Clusters of different sizes were used in the modelling and the best agreement between calculated SERS values and the experimental data was established for Cu_20_-Py.

In [34], DFT calculations showed that procainamide molecules are chemisorbed on the rough surface of AgNPs mainly at the double binding sites of C=O and NH_2_.

**Figure 7.**
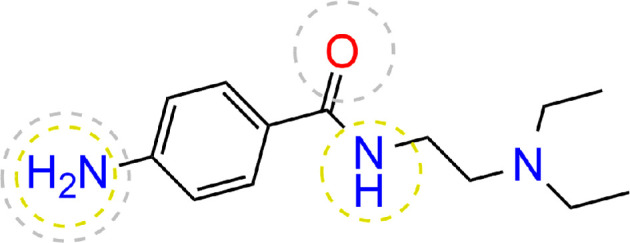
Procainamide adsorption sites. Functional groups involved in adsorption are labelled: grey circles indicate interaction sites with AgNPs (silver nanoparticles); gold circles indicate interaction sites with AuNPs (gold nanoparticles).

However, subsequent studies [35] with gold nanoparticle showed that procainamide adsorbs through HN- and H_2_N- groups (figure 7).

**Figure 8.**
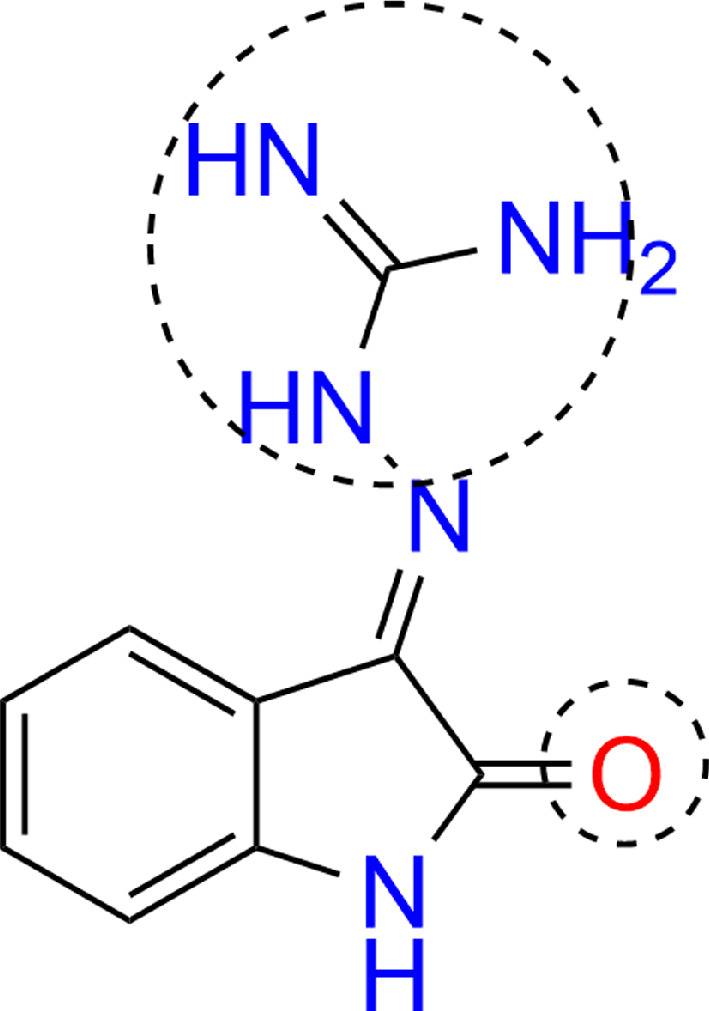
OHC adsorption sites. Functional groups involved in adsorption are labelled.

One study [36] investigated the use of nanoparticles for novocaine detection; and DFT calculations confirmed that the molecule adsorbed reasonably well on the surface of gold nanosphere. In [37], the characteristic Raman peaks of a silver nanocluster with aflatoxin B1 were determined by comparing the results of DFT calculations. Research in [38] showed that (Z)−2-(2-oxoindolin−3-ylidene) hydrazine−1-carboximidamide hydrochloride (OHC) adsorbed on an Ag_6_ silver cluster with a binding energy of −6.57 eV through the N(C=NH) and H(NH) atoms. This was evidenced by the fact that when the Ag_6_ cluster was introduced into the system, the electron-rich region around the electronegative double-bonded O and N(C=NH) atoms of OHC decreased, indicating the chemisorption of OHC on the nanoparticle surface and charge transfer from OHC to Ag cluster (figure 8).

In [39], Raman spectra obtained experimentally and by DFT for six fentanyl preparations were analysed to identify individual markers. A particularly interesting paper [40] provided a more detailed investigation of the chemical amplification process in SERS, using piperidine’s interaction with a silver surface as a case study. As observed in the works listed above, differences between the normal Raman (nR) spectrum and the SERS spectrum of the molecule were also present here. The authors analysed these spectral features using DFT calculations considering model complexes with piperidine molecules in two possible conformational situations: axial and equatorial, associated with a neutral or positively charged silver atom. The possibility of deprotonation of piperidine upon adsorption on a positive metal surface was also taken into account. The fact that silver Ag(I) ions activated by chloride ions were most likely present on the surface of the nanoparticle was also taken into account. DFT calculations revealed that the complex formed by the deprotonated molecule in the equatorial conformation associated with the silver ion was formed on the surface of the particle, which is characterized by charge transfer from piperidine to metal. This highlights one of the mechanisms in the chemical enhancement of SERS.

**Figure 9.**
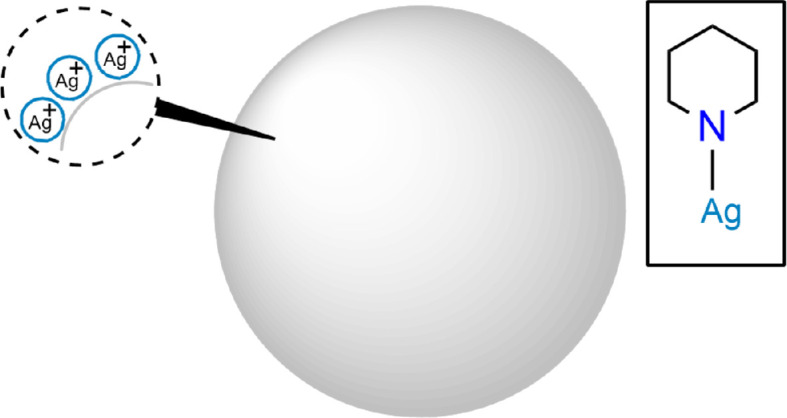
Silver ions on the surface of nanoparticles.

The study also described another mechanism of chemical amplification—namely, the resonance effect in Raman light scattering. The effect occurred when the Raman waves hit the electronic excitation zone of the molecule/metal complex. Further modelling showed a strong excitation band in the green region of the spectrum, which was fully attributed to the HOMO–LUMO transition. This finding indicates that the resonance between the excitation radiation and the absorption band of the complex is responsible for the enhancement effect (figure 9).

**Figure 10.**
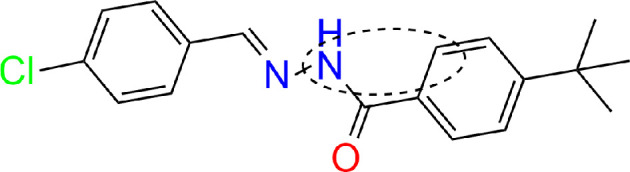
TCB adsorption sites. Functional groups involved in adsorption are labelled.

In [41], the interaction of amphetamine (AMP) and its derivatives with the surface of gold and silver nanoparticles was studied. DFT calculations revealed that AMP binds to the nanoparticle through the interaction of the aromatic ring perpendicular to the surface of the gold nanoparticle, as indicated by the enhancement of the corresponding peaks in the spectrum. The same binding pattern was observed for methamphetamine (MET). However, for methylenedioxymethamphetamine (MDMA), the interaction occurs through the oxygen atom in the structure of the compound, while still maintaining the perpendicular orientation relative to the surface of the nanoparticle. For silver nanoparticles, the authors suggested that the interaction of all three compounds most likely occurs through the amino group. At the same time, they emphasized multiple discrepancies between the modelled and the experimental spectra. In [42], the interaction of (E)-tret-butyl-N-(4-chlorobenzylidene) benzyhydrazide (TCB) with Ag was examined. By modelling and comparing theoretical and experimental spectra, the researchers established that the molecule adopts a tilted orientation with respect to the nanoparticle, and adsorption occurs through the possible binding of N10 and C2 atoms to Ag. The red shift in UV-Vis absorption indicated the presence of TCB molecules adsorbed on Ag, while FMO analysis demonstrated charge transfer between the metal cluster and the molecule. The charge transfer interaction was further confirmed on the molecular electrostatic potential (MEP) surface (figure 10).

**Figure 11.**
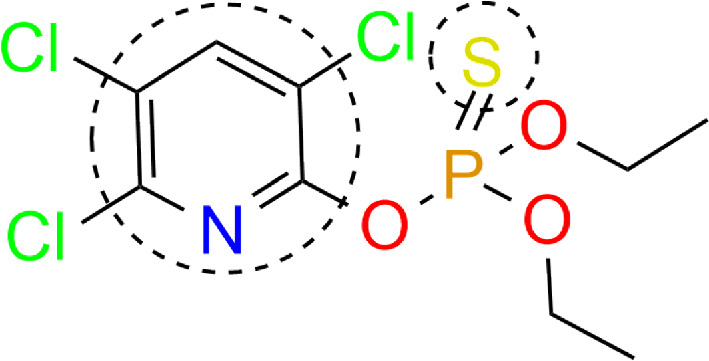
Chlorpyrifos adsorption sites. Functional groups involved in adsorption are labelled.

In [43], the influence of halogens in meta- and para-positions in benzolthiol on the chemical enhancement mechanism in SERS was investigated. The study found that, for para-substituents, the chemical mechanism enhancement factor decreases with increasing atomic number of the halogen, whereas no such tendency was detected for meta-substituents. In another study [44], the interaction of chlorpyrifos (CPF) with silver nanoclates was examined. The geometry of the ligand–cluster interaction showed that the CPF molecule is mainly adsorbed on the surface through the S atom, through the pyridine ring involving Ag-S covalent coordination, and through van der Waals physical adsorption. This behaviour can be attributed to the CPF’s electron-donating properties during adsorption. Specifically, CPF gives up an electron from its lone pairs on S and Cl atoms, as well as a π-electron on the S=P bond, to the silver atoms on the surface. After that, the positive charge of the silver surface is shifted to the CPF fragment via Ag-S and Ag-Cl (figure 11).

**Figure 12.**
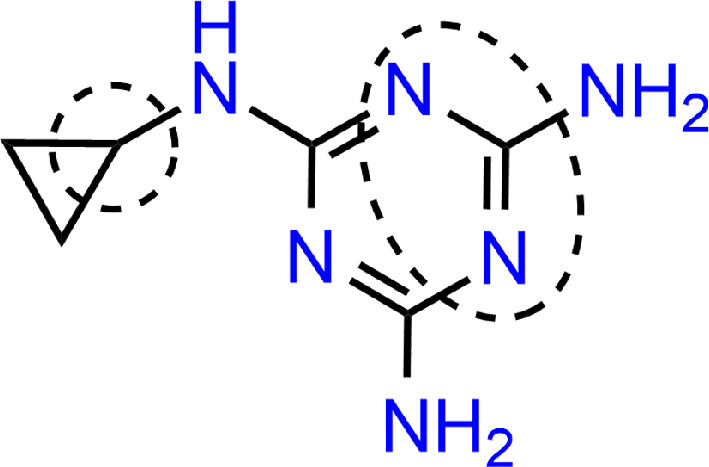
Cyromazine adsorption sites. Functional groups involved in adsorption are labelled.

Another work [45] looked at the interaction of the fungicide thiram (THR) with silver nanoparticles. Analysis of the complex showed that THR interacts with the silver cluster through two S atoms in the upper part of the cluster, and adsorption occurs spontaneously, as evidenced by the negative Gibbs energy value. In addition, electronic properties of the complexes were analysed to reveal that the electron density is transferred from the adsorbate to the Ag_20_ cluster in the ground state. In [46], the interactions of 2,4,5-trichlorophenoxyacetic acid (2,4,5-T) molecule with small clusters of silver (Ag_*n*_, where *n* = 4, 8, and 20) were calculated using DFT. The results indicate that the most stable adsorption configuration is formed by coordinating the Cl centres and carbonyl group C=O to the Ag atoms on the surface. Analysis of the charge transfer mechanism and boundary orbital distribution shows electron transfer from 2,4,5-T to the ground state cluster, while the opposite trend is observed for the excited singlet state process, which subsequently leads to chemical enhancement of the SERS signals. A study [47] of bisphenol (BPA) and its monohydroxylated derivative investigated their interactions with nanoparticles. Electronic structure calculations of various Ag_2_-BPA and Ag_2_-BPA(OH) complexes, along with their calculated conventional and resonance Raman spectra in the first excited electronic state, indicate that surface plasmon-like resonance within the silver cluster is responsible for the enhancement of bands corresponding to physically adsorbed forms of BPA(OH). Meanwhile, the charge transfer enhancement mechanism was ruled out, with the intramolecular resonance transition localized within the phenolic framework. Additionally, BPA(OH) interacted with the metal surface through two oxygen atoms as well as the inner face of the aromatic ring. The mechanism of chemical adsorption of icotinib on AuNP gold nanospheres [48] was explained using a gold cluster of six Au_6_ atoms in the modelling. The calculations showed that icotinib has strong electronegativity near the acetylene group, two N atoms of quinazoline ring and the O atom of tetraoxycyclododecyl, suggesting that icotinib adsorbs on the AuNPs surface through these four sites. The binding energy calculation further confirmed that the complexes formed by the chemical energy of the ethynyl group are the most stable. The molecular boundary orbitals of icotinib and icotinib-Au_6_ indicated that the molecular energy gap decreases with the introduction of Au_6_ clusters. At the same time, charge transfer was primarily distributed over the ethynyl group, quinazoline and benzene ring of icotinib. A comparison of the Raman activity spectrum of icotinib and icotinib-Au_6_ revealed the phenomenon of selective enhancement of spectral peaks. The amplification effect of this phenomenon was attributed to the charge transfer effect between the molecule and the metal, which enhances the polarizability of the molecule and increases the Raman cross-section of the molecule, resulting in the increase of the Raman spectrum peak. In another study [49], the system of interaction between cyromazine and gold nanosphere was analysed. The results showed that the energy gaps of cyromazine-AuNPs complexes decreased, and the chemical activity increased. Electron transfer from cyromazine to AuNPs was also observed, which was further supported by the change in polarization. On Au_6_ and Au_20_ clusters, the molecule was adsorbed through the atomic N2, N4 rings of triazine and H13 in cyclopropyl to form stable complexes with AuNPs. Additionally, modelling revealed that adsorption strength increased with larger gold cluster sizes (figure 12).

The interaction of 5-fluorouracil (5FU) with gold clusters was investigated in [50]. Analysis of the SERS spectra of 5FU adsorbed on the Au surface showed that the valence vibrations of N-H and C=O bonds play a primary role in the SERS phenomenon. The analysis of the frontier molecular orbitals (FMOs) showed that the main component of the 5FU–Au interaction is charge transfer from the molecule to the gold clusters. DFT modelling in [51] demonstrated that adsorption of thiobenzoic acid on colloidal silver nanoparticles occurs through the sulfur atom (monodentate) of its anionic moiety. Similar to other aromatic amides, thiobenzamide also adsorbs in deprotonated form on the silver surface. The corresponding azanion binds to the metal via the sulfur and nitrogen atoms of the ionized thiocarboxamide group (bridging bidentate). A study of Triadimenol orientation on the surface of silver nanoparticles [52] showed that the triazole group in the compound interacts directly with the Ag surface, while the 4-chlorophenyl group is close but at an inclination relative to the Ag surface, and the 3,3-dimethylbutan−2-ol group is positioned away from the surface.

In [53], DFT calculations of the alcannin and shikonin dyes allowed us to attribute the experimental Raman and SERS bands to the normal vibrational modes of these dyes. The Raman spectra of alcannin and shikonin closely resemble their SERS spectra at acidic pH, suggesting that the dyes exist in a neutral form on Ag nanoparticles under these conditions. However, several spectral differences indicate that the molecules adsorb perpendicularly on the surface of AgNPs via nearby carbonyl and hydroxyl groups in the rings, together with the OH side chain. At neutral pH, the SERS spectra of red dyes differ from those at acidic pH, which suggests deprotonation of the OH-group. The interaction of the ionic varieties of alkanine and shikonine with the Ag surface is equivalent to the neutral forms of the dyes. Comparing the SERS spectra of both dyes at acidic and neutral pH revealed several differences.

In [54], a study of SERS spectra showed that the chemisorption of cefoperazone on gold and silver nanoparticles occurred through the nitrogen ring, while the changes observed in the spectra recorded at different concentrations were attributed to the reorientation of the adsorbed particles relative to the metal surface. The adsorption mechanism of 5-bromo-N-[4-bromo−3-(trifluoromethyl)phenyl]−2-hydroxybenzamide (BTB) was examined in [55], showing that it binds to the silver nanoparticle through O, F and Br atoms while maintaining an inclined orientation relative to the metal surface. The HOMO–LUMO energy difference for BTB alone and in complex with a silver cluster equalled 4.66 and 2.84 eV, respectively, with the decrease confirming adsorption. The electrophilicity index values of BTB and BTB-Ag_6_ systems were, respectively, 3.72 and 4.47 eV. The electrophilicity index of BTB-Ag_6_ is higher than that of BTB molecule, indicating that BTB tends to become more electrophilic upon addition of Ag_6_ metal cluster, facilitating electron capture.

DFT calculations in [56] were used to optimize and analyse the molecular structures of (E)−4-methoxy-N'-(2-(trifluoromethyl)benzylidene)benzohydrazide (EMT) and EMT-Ag_6_ systems. Structural changes indicated EMT adsorption on Ag_6_. Peaks in the UV–Vis spectra of EMT, which show red shift and intensity decrease in the presence of Ag_6_, further confirmed this interaction. FMO analysis was used to evaluate the charge transfer interaction and reduced energy gap values, indicating chemisorption of EMT on Ag_6_. The charge transfer from Ag to EMT was verified using MEP and Malliken charge analysis. The results were further validated by nR and SERS modelling, which provided evidence for the chemisorption of EMT on the metal surface. A number of nR modes become active in the SERS spectrum as a result of the change in polarizability by varying the concentration of colloidal silver nanoparticles as well as the orientation of EMT with Ag. It was shown in [57] that the adsorption of lipoic acid (LA) on the surface of silver nanoparticles goes through the carboxyl group and is predominantly perpendicular to the metal surface. Moreover, at concentrations below 10^−7^, the adsorbed LA molecules change their orientation relative to the metal surface and adopt a more slanted going on parallel configuration relative to the Ag nanoparticle surface. Modelling of alizarin (AZ) adsorption on silver nanoparticles in [58] accounted for the presence of non-reduced silver ions. In addition, the study examined deprotonated dye molecules at different pH values.

In [59], it was found that 6-mercaptopurine at pH = 4.5 assumes a planar orientation when adsorbed on the surface of a gold nanosphere. A possible binding mechanism involves sulfur and N7/N1 atoms with the contribution of π-interaction of the pyrimidine ring. The study in [60] compared the Raman spectra of Ala-Trp dipeptide adsorbed on gold nanospheres, which were prepared by two different methods: the citrate method, where sodium citrate was used as a reducing agent (denoted as ST); and the reduction with sodium borohydride (denoted as BH). Ala-Trp was mainly adsorbed through the amino group of Ala residue on CT gold nanoparticles, where the perpendicular orientation of the indole ring to the surface is observed. However, for BH nanoparticles, Ala-Trp adsorbed through the π-electrons of the indole ring, with a planar orientation of the indole ring towards the surface. The change in pH in gold colloids also confirmed different adsorption mechanisms of Ala-Trp with colloidal gold nanoparticles. This is supported by the change in the surface orientation of Ala-Trp evidenced by the strong appearance of amide bands with CT gold colloids and the variations in the orientation of the indole ring relative to the surface with BH colloidal gold nanoparticles.

In [61], the interaction of silver nanoparticles with drug molecules (sulindac) was analysed. A significant increase in intensity was obtained in Raman light scattering bands at 1111, 1653, 1607 and 1340 cm^−1^, which corresponds to -S=O stretching, -C=O stretching, -C=C- stretching and ring stretching vibrations, respectively.

**Figure 13.**
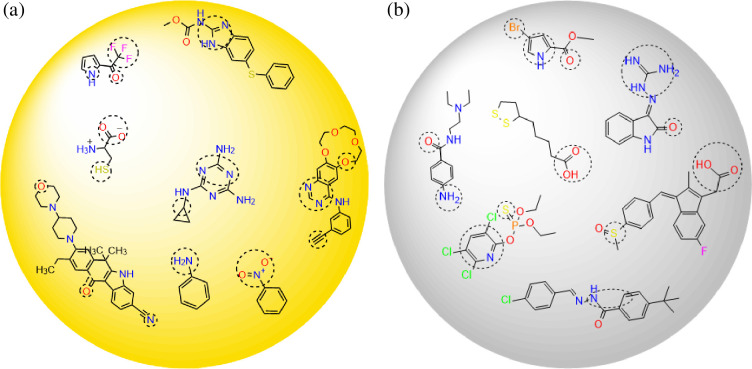
Gold (a) and silver (b) nanoparticles and adsorbed molecules through atoms and functional groups according to DFT calculations.

A method for synthesizing silver nanoparticles using glycose was demonstrated in [62]. SERS and DFT calculations were used to analyse the isomers of D-glucose and D-glucanate anion on the surface of these nanoparticles. Experimental and theoretical data confirmed adsorption of the molecules on the nanoparticle surface. In this case, D-glucanate anion formed the most stable complex with an adsorption energy value of 1.93 eV, while α-d-glucose and β-d-glucose molecules differed slightly in stability. Additionally, it was found that α-d-glucose is most likely adsorbed through two oxygen atoms, which is not observed in the β-isomer due to their locations in opposite sides. Building on this research [63], employed glucose-derived nanoparticles to detect the antibiotic oxytetracycline. Vibrational frequencies at 596, 780, 797, 865 and 1015 cm^−1^ characteristic of the antibiotic were identified as a result, with theoretical calculations suggesting that adsorption of oxytetracycline occurs predominantly through hydroxyl groups (figure 13).

## 3. Effect of cluster size and cluster composition in modelling on the density functional theory result

**Figure 14.**
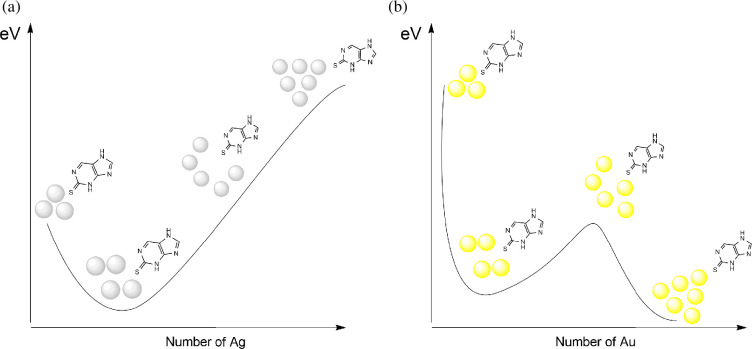
Energy gap of 3,7-dihydropurine−2-thion complexes with silver (a) and gold (b).

A crucial aspect of modelling the interaction between a molecule and a metal nanoparticle is the accurate construction of the nanoparticle itself, including selecting the appropriate geometric structure. The use of a single metal atom is insufficient to reflect the full characteristics of the NP-molecule complex. Thus, in the above-mentioned [27], the dependence of the change in the energy parameters of the complex on the size of the cluster was analysed. For gold, the energy parameters varied with the number of atoms in the cluster, whereas no such dependence was observed for silver (figure 14).

The choice of crystal lattice for NP is also essential for more detailed modelling. In [64], a comprehensive analysis was conducted on six lattices of gold, silver and bimetallic nanoparticles (Au_20_Td, Ag_20_Td, Au_20_Cs, Ag_20_Cs, Au_20_Cb1 and Ag_8_Au_12_Cb2). The study demonstrated how the adsorption site of the pyridine molecule (Py) is affected on the V vertex and on the S face. For gold nanoparticles, the denser Cs and high-semitic Cb lattices exhibited similar energy characteristics, while the tetrahedral Td lattice had lower energy. Both Cs and Cb geometries were found to be stable. In contrast, for silver nanoparticles, the minimum energy values for Td and Cs lattices were close, indicating that both geometries are possible. Cubic structures (Cb1 and Cb2) were very symmetric, with Cb1 forming an octahedral (Oh) structure and Cb2 a cube with an internal icosahedron. The Ag_8_Au_12_ bimetallic cube retained the inner icosahedron, whereas the Au_20_ monometallic cube turned into an Oh structure. At the same time, the opposite Au_8_Ag_12_ cluster lost its structure and entered a transition state. Chemical enhancement, in the form of static or chemical binding and as a charge transfer mechanism, was observed in the Au_20_Cb1-Py-V structure. In the Me_20_Cs and Ag_8_Au_12_-Py bimetallic cases, static enhancement by an order of 10 was reported, with a low percentage of charge transfer excitations in the 500−530 nm window. Nevertheless, the gain factors obtained in the Cs and Cb structures exceeded those for the Td geometries at both V and S positions. Building on this work, the same authors [65] modelled the structure and parameters for silver, copper and bimetallic cluster M_10_N_10_ (M = Ag, Cu) of tetrahedral structure Td (Td, Td1 and Td2) in complex with Py. All clusters were stable and the stability was also related to their deviations from Td symmetry. Ag_10_Cu_10_ Td2 and Cu_10_Ag_10_ Td1 were the most favourable clusters from the electronic and structural point of view. As in the previous paper, binding sites at vertices V and faces S were also analysed. The calculations showed that the gain at the Py-V vertex was higher than at the Py-S face. The static SERS response for the Cu-Py-V transition was 5–10 times greater than the EF of Ag-Py-V and up to 28 times greater than the EF of Py-S complexes. The Raman spectra of Ag-Py-V or -S bimetallic clusters Td1 and Td2 were enhanced compared with monometallic clusters, indicating that adding Cu atoms could improve the Ag_20_Td cluster. For the analysed complexes, the following order of decreasing enhancement factor was established: Cu-Py-V > Ag-Py-V > Cu-Py-S > Ag-Py-S. The scattering efficiency of the Ag-Cu bimetallic cluster was also confirmed in [66]. The study found that adding up to five Cu atoms to the Ag_8_Td cluster maximized the intensity of the Raman peak of the Py adsorbate by a factor of 170. At the same time, a higher Cu/Ag ratio did not further strengthen the enhancement. A similar trend was also observed for pyrazine and 3H-pyrrole ligands. This spectroscopic enhancement phenomenon can be attributed to a marked increase in charge transfer from the mixed cluster to the Ru ligand.

**Figure 15.**
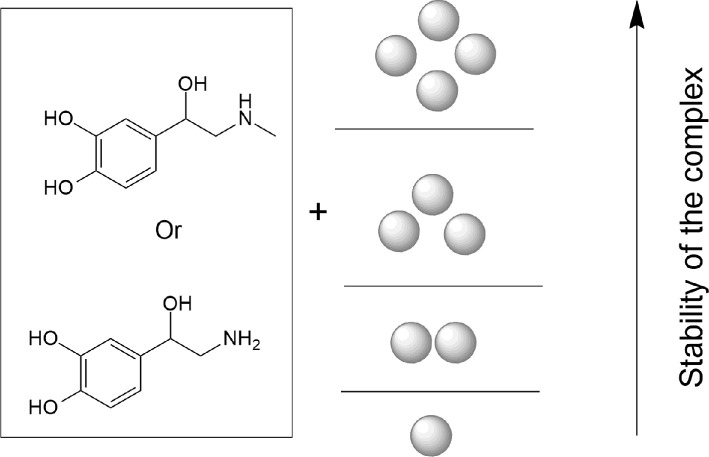
Dependence of the stability of the complex on the number of atoms in the cluster of the model.

In [67], a static mechanism of chemical amplification for norepinephrine in combination with a silver nanoparticle was observed. As the number of silver atoms in the cluster increased (from one to four), so did the intensity of some peaks. It was also shown that the increase in Raman intensity was associated with an increase in the global electrophilicity indices (ω), static average polarizability (α_0_) and total polarizability (Δα). In addition, the calculations showed that the stability of the complex increased with the increase in the number of atoms. Later, a similar work examined adrenalin adsorption [68], showing that the calculated Raman activity also increased with increasing silver cluster size. It was shown that the increase in Raman activity was associated with an increase in static average polarizability, total polarizability and global electrophilicity index, the value of which changed with increasing cluster size (figure 15).

In another study [69], bimetallic clusters were constructed with formulae Cu_4_-xMx and Cu_13_-yMy, where M = Au or Ag, x = 0−4, and y = 0, 1, 3, 4, 6, 7, 9, 10 or 13. The adsorbed molecule was 4-nitrothiophenol (4-NBT). Calculations showed that the introduction of a small amount of Cu at the surface of Ag nanoparticles and in the core-shell structure gave the best enhancement result. Сlusters of Cu_2_Ag_2_, Cu_1_Ag_12_, Cu_1_Au_12_, Cu_3_Ag_10_, Cu_3_Au_10_, Cu_14_Ag_9_, Cu_4_Au_9_, Cu_9_Au_4_ and Cu_10_Au_3_ showed enhanced Raman light scattering, whose diffraction peak intensity exceeded that of the 4-NBT molecule on Ag_4_, Ag_13_ and Au_13_ clusters by over 11%.

In [70], indium (In) was explored as a metal for SERS. Several In(n) clusters with n ranging from 2 to 20 atoms were modelled. The results confirmed that the In8 was the most stable cluster configuration. Although the stability of indium clusters generally increased with the increasing number of atoms, starting from In_11_ the energy values became less and less different, with In_9_ cluster being the least stable structure compared with other optimized clusters. Another study [71], modelled the interaction of benzothiazole (BTH) with star-shaped copper, silver and gold nanoparticles containing 12 atoms. According to the results, the molecule exhibits the highest adsorption energy with copper nanoparticle with a value of −17.57 kcal mol^−1^. It was also found that the interaction of nanoparticles of all three metals with BTH resulted in the charge transfer, introducing an additional state near the Fermi level. In addition, a change in the polarizability of the molecule near the clusters was recorded.

In [72], a pyridine (PYR) complex with another bimetallic cluster PYR-Ag_x_-M_y_ (x = 4/5, y = 2/1 and M = Au/Ni/Cu) was analysed. Calculations showed that Ag_4_Au-PYR and Ag_5_Ni-PYR were the most stable systems. While Ag_5_ clusters showed similar interaction energies across different metals, Ag_4_ clusters exhibited significant energy variations. In addition, it was found that the non-covalent interactions between Ag_4_-Ni_2_ and PYR system were strong, with electron localization over the PYR residue in the cluster, which could be observed from the electron localization function and localized orbital locator analysis. Interestingly, while increasing the size of silver or gold clusters improved system stability, for copper clusters, the opposite was true. The most stable cluster was Cu_4_, as shown in [73]. Nonetheless, as with other metals, increasing cluster size led to higher mode intensities in the SERS spectra.

## 4. Conclusions

DFT calculations serve as a reliable tool for investigating the mechanisms of SERS amplification. As practice has shown, in many cases, the theoretical predictions closely align with experimental data, allowing for the development of a comprehensive theory describing molecular interactions with nanoparticles (NPs). These calculations help determine key factors such as the functional groups involved in NP binding, the analyte’s conformation and how molecular orientation changes with concentration. However, certain factors require special attention, particularly the cluster size that is used in model construction. As demonstrated in the studies reviewed, some interesting conclusions can be drawn about the change in the energy gap, the value of which changes with increasing number of atoms in the cluster, even if the change does not always correlate with the cluster size. In addition to the size of the cluster, the shape and crystal structure of the NP can play a significant role. When embedded with atoms of other metals, forming bimetallic complexes, NPs can achieve even greater SERS signal amplification. Moreover, there is an assumption that metal ions present on the NP surface contribute to the chemical mechanism of amplification. It sometimes happens that constructed models of the same molecules with clusters of different metals can differ in how closely the theoretical calculations agree with experimental data. Assuming that all calculations are accurate, one possible explanation is the occurrence of photochemical reactions in these complexes, though further research is required to confirm this [74].

## Data Availability

This article has no additional data.
